# A qualitative assessment of provider-perceived barriers to implementing family-based treatment for anorexia nervosa in low-income community settings

**DOI:** 10.1186/s40337-024-01008-w

**Published:** 2024-04-25

**Authors:** Amy H. Egbert, Bailey Irizarry, Elisabeth Lualdi, Christina C. Tortolani, Deidre L. Donaldson, Andrea B. Goldschmidt

**Affiliations:** 1 Department of Psychiatry and Human Behavior, Warren Alpert Medical School of Brown University, Providence, RI USA; 2 Department of Psychological Sciences, The University of Connecticut, Storrs, CT USA; 3 School of Public Health, Brown University, Providence, RI USA; 4 Department of Counseling, Educational Leadership, and School Psychology, Rhode Island College, Providence, RI USA; 5 Department of Family Medicine, Warren Alpert Medical School of Brown University/Gateway Healthcare, Providence, RI USA; 6 Department of Psychiatry, University of Pittsburgh, Pittsburgh, PA USA

**Keywords:** Anorexia nervosa, Community settings, Adolescents, Implementation, Low-income

## Abstract

**Background:**

Family-based treatment (FBT) is a front-line empirically supported intervention for adolescent anorexia nervosa, but it is often inaccessible to families from lower income backgrounds, as it is most typically available in specialty research and private practice settings. In preparation for a pilot trial of FBT delivered in the home setting, this study qualitatively examined provider perceptions of implementing FBT in lower-income communities.

**Methods:**

Eating disorder clinicians working in community clinics (therapists, medical doctors, dietitians, and social workers; *n* = 9) were interviewed about their experiences using FBT. Interview transcripts were analyzed both deductively, using an approach consistent with applied thematic analysis, and inductively, using the Replicating Effective Programs implementation framework, to examine barriers to FBT implementation.

**Results:**

Prevailing themes included concern about the time and resources required of caregivers to participate in FBT, which may not be feasible for those who work full time, have other caregiving demands, and/or lack family support. Psychosocial problems outside of the eating disorder, such as food insecurity, other untreated mental health concerns (in themselves or other family members), or externalizing behaviors on the part of the adolescent, were also discussed as barriers, and participants noted that the lack of cohesive treatment teams in the community make it difficult to ensure continuity of care.

**Conclusion:**

Findings from this qualitative study indicate the need to address systemic socioeconomic barriers to improve the efficacy of implementation of FBT in the community and to understand how provider perceptions of these barriers influence their uptake of FBT.

## Abstract (plain language summary)

This study looks at reasons why providers think that family-based treatment for adolescent anorexia nervosa (FBT) is difficult to implement in community settings serving families from lower-income backgrounds. When interviewed, participants expressed concerned that FBT requires a lot of time and resources from families and that problems beyond the eating disorder (like having other mental health conditions or not having enough money to make ends meet) get in the way of treatment.

Anorexia nervosa (AN) is a severe psychiatric illness that disproportionately affects adolescents and young adults and is associated with mortality rates that are 5 to 10 times that of the general population [1, 2]. Although it was historically thought that AN primarily affected individuals from wealthier backgrounds, a growing body of evidence suggests that eating disorders occur in individuals from a diverse range of socioeconomic groups [3]. Specifically, prevalence rates of AN appear to be rising in youth from lower income backgrounds [4]. Despite this rise, lack of access to high quality specialty care and cost constraints associated with treatment engagement make it difficult for youth from lower income backgrounds to receive the care that they need [5]. Recent data suggest that youth with public insurance are one-third less likely to receive the recommended treatment for their eating disorder than those with private insurance, and this is compounded for youth from Black or Hispanic backgrounds [6].

Family-based treatment (FBT) is an empirically-supported intervention for adolescent AN [7, 8]. The core focus of FBT is to empower parents and caregivers to lead the adolescent’s recovery by taking charge of the eating behaviors of the adolescent in a phasic approach, including: Phase 1, which focuses on weight restoration; Phase 2, which focuses on returning control of eating back to the adolescent; and Phase 3, which seeks to establish healthy adolescent functioning outside of the eating disorder and to expand the relationship between parents and adolescent beyond the eating disorder [9]. Although FBT is recommended as the front-line treatment for adolescent AN [10], it is often inaccessible to families from lower income backgrounds due to its limited implementation outside of specialty research and private practice settings [11]. Compounding this problem, less than 5% of certified FBT clinicians are contracted with Medicaid, making it difficult for many families to afford treatment [5]. Because of this, recent efforts are underway to partner with community medical clinics and mental health centers to deliver FBT to families who need treatment [12, 13].

Our group is currently conducting a pilot effectiveness-implementation trial testing the delivery of FBT-informed treatment in the home setting (FBT-HB) by training community therapists and partnering with clinics that serve patients from lower-income, primarily racial and ethnic minority backgrounds [13]. Although the specific parameters vary by state and health insurance requirements, home-based treatments are typically delivered as intensive bursts of treatment (i.e., multiple sessions per week) in a relatively short period of time (3–6 months) and can be conceptualized as an intermediate level of care between outpatient and hospital-based care. A home-based model of care has not been historically applied to the treatment of AN, but FBT-HB may help address the need for increased accessibility to eating disorder treatment for youth from lower income backgrounds by eliminating logistical barriers such as transportation concerns and using community agencies to provide wraparound care to meet the needs of families that have multiple stressors aside from the eating disorder. Primary modifications made to FBT to adapt it to a home setting include providing a more intensive but shorter-term treatment (i.e., more hours per week but fewer weeks of treatment in total), multiple family meals over the course of treatment, and therapist help with providing and supervising meals along with parents [14]. Therapists may also attend medical appointments related to the eating disorder and visit schools to aid with supervised meals, when necessary. However, the phases and goals of traditional FBT (i.e., first empowering parents for weight restoration and then transitioning toward returning appropriate control back to the adolescent) remain the same for FBT-HB.

Although there is a growing body of literature examining therapist perspectives on the overarching challenges of conducting FBT, most have focused on specific components and mechanisms of the treatment itself (e.g., weighing the adolescent, fostering parental empowerment) that impact general acceptability and feasibility [15–17]. However, much less is known about whether providers perceive specific barriers to implementing FBT with families from lower income backgrounds. For example, a recent study by Dimitriopoulos and colleagues examined therapist perceptions of delivering FBT to families from “diverse backgrounds” [18]. Although the definition of “diverse” included individuals from lower income backgrounds, it also included individuals from a wide variety of other diverse backgrounds (e.g., race, ethnicity, gender, sexual identity, trauma history), making it difficult to know whether certain challenges might have related more to individuals from lower income backgrounds or to those with other diverse identities. Furthermore, despite the need to have buy-in from providers from all aspects of the treatment team (e.g., medical providers), most studies have only included therapist perspectives, and those that have examined FBT implementation from a multidisciplinary perspective have mostly focused on organizational factors relevant to FBT implementation [19]. In the present study, we collected perspectives from therapists, medical providers, nutritionists, and social workers about their experience using FBT to serve families from the community. We aim to use the information gathered in these interviews to inform the delivery of FBT-HB given the context of our specific setting (low-income, predominantly racial and ethnic minority populations seeking care through community agencies).

## Method

### Participants

We conducted interviews with nine providers involved in treating adolescents with AN in the northeastern United States. This included a mixture of therapists, medical providers (i.e., MD or NP), registered dietitians, and social workers. All participants identified as female, a majority identified as non-Hispanic white, and the others identified as being Hispanic or biracial. Five participants worked in specialty eating disorder clinics that focused on medical management of eating disorders, and four worked in community mental health or medical clinics. All were recruited for the study from organizations with which the study team had established community partnerships based on their involvement in making referrals or providing care for families engaged in the pilot open trial. All participants either had experience delivering FBT-HB or working in consultation with others who had delivered FBT (in the case of non-therapist participants). Therapists were trained in traditional outpatient FBT by certified experts in the field and they all had experience delivering FBT in the home setting as part of the feasibility testing that we conducted prior to beginning the formal pilot trial of FBT-HB. They were also invited to participate in our formal pilot trial of FBT-HB. The other participants (i.e., medical providers, dieticians, social workers) provided medical, nutritional, and case management to patients with restrictive eating disorders. These participants all had experience collaborating with therapists delivering FBT either in a traditional outpatient setting or as a part of FBT-HB feasibility testing. They have also made up our primary referral source for the FBT-HB pilot trial.

### Procedure

Guided by principles of the U.S. Centers for Disease Control and Prevention Replicating Effective Programs Implementation Strategy (i.e., REP), we sought to conduct a series of qualitative interviews with community providers who were typically a part of the multidisciplinary eating disorders team for the patients who would be the target of the FBT-HB intervention in the community agencies. The REP framework was designed to bridge the gap between research and practice by packaging interventions in such a way that they can be implemented in non-academic settings within the community [20]. It has four phases: pre-conditions, pre-implementation, implementation, and maintenance and evolution. Our qualitative interviews were conducted as a part of pre-conditions phase of REP, which seeks to understand the need for a new intervention in the setting in question prior to implementation, ensure that the intervention fits local stakeholder priorities, and identify barriers to implementation [20]. We chose to focus on provider perspectives because of their role as the gatekeepers of treatment, as research suggests that if providers have negative beliefs about the feasibility or acceptability of a particular treatment, even if it is evidence-based, they are less likely to implement it or recommend it to their patients [21, 22].

Study participants were invited to participate in one-on-one semi-structured interviews conducted over Zoom by A.E., a PhD level psychologist with expertise in FBT and training in qualitative methods and E.L, a bachelor’s level research assistant trained in qualitative methods. Interviews lasted approximately 60 min. Demographic information was collected verbally at the beginning of the interview. The example interview questions (see Table 1) were geared toward elucidating each participant’s understanding of the principles of FBT, their opinion about the barriers and benefits of implementing FBT inside or outside of the home setting, and their general impressions of delivering eating disorder treatment and supporting FBT in the communities with which they worked. Because of the diverse clinical experience of the participants, we focused less on their thoughts about the setting in which they implemented FBT (e.g., the home) and more on their perceptions of the feasibility of achieving the primary goal of both FBT and FBT-HB, having caregivers assume primary responsibility for addressing the adolescent’s eating disorder to achieve weight restoration, in the communities with which the participants worked. This broader focus was also necessary given that many participants switched from in-person care to virtual treatment during the COVID-19 pandemic. During the time of the interviews, most participants had switched back to fully in-person care or were providing a hybrid model, so their experiences also reflected these challenges with the modality of treatment. Aside from the family and structural level barriers that participants identified as being problematic for implementation of FBT, we also asked participants to comment on barriers that made it difficult for the treatment team to function efficiently.

**Table 1:** Qualitative interview questions

Section	Questions	Notes
1. FBT Basics	How would you describe FBT?	
	What would you describe as the “key ingredient” of FBT?	Try to obtain a sense of why they think FBT works when it works
	What are the pros and cons of the FBT model?	
2. Implementing FBT	What is your perception of the characteristics that make a family more or less likely to succeed in treatment?	
	What were the barriers and successes to implementing FBT in the community in which you work?	
	How has it been consulting with a multidisciplinary team of providers?	
3. Looking Forward	If you had all the time and money in the world, what treatment would you use for eating disorders?	If needed, probe for whether they would create a new treatment entirely or if they would adapt a current treatment (like FBT) to get at monetary/practical barriers that they see
	Would you use FBT again? Why or why not?	Follow-up questions may relate to asking which populations they might recommend for FBT and which populations they would not recommend for FBT.

### Data analysis

Interviews were audio recorded and transcribed verbatim by a member of the study team. They were then de-identified and transcripts were imported into NVivo, a qualitative data management software. Data were analyzed using an approach consistent with applied thematic analysis, and we used a hybrid approach of both inductive and deductive code formation. An initial coding scheme was created using the following steps: (1) interviews were read for familiarity, (2) codes were generated deductively guided by the implementation framework of REP (e.g., barriers, setting specific concerns), (3) new codes were created inductively based on repeated concepts in the data after a second read of interviews. All interviews were double coded by the first author and a second member of the study team (E.L. and B.I.). Both coders carefully reviewed each transcript for the presence of the relevant codes. Any discrepancies or emergence of new codes were discussed by both coders until consensus was reached. The first author then analyzed the codes for prevailing themes and subthemes and shared that paradigm with the second coder who independently reviewed it to ensure that themes and subthemes appropriately represented the original transcripts. Discrepancies were again reviewed, and themes were collapsed, edited, or removed until consensus was reached.

Sample size was determined by monitoring data saturation throughout the interview process through discussion among the study team. We considered data saturation to be achieved when no new information regarding barriers to implementing FBT in families with lower incomes was obtained. Following detection of saturation, interviews were continued in order to include at least one provider from each of the multidisciplinary roles on the teams that we worked with (i.e., MD, NP, therapist, social worker, registered dietitian). This left us with *n* = 9 interviews, which is consistent with recent research suggesting that 6–7 interviews can often capture the majority of themes in a homogenous sample [23].

## Results

### Qualitative themes

The overarching themes identified included lack of family time and resources to adhere to the FBT model, psychosocial barriers unrelated to the FBT model that interfered with its implementation, and organizational barriers to implementation (see Table 2). Although the first two themes were generated inductively through thematic analysis of the data, we specifically queried participants about organizational barriers to FBT implementation to leverage their role as a part of organizations that we have been working with since initial conceptualization of the trial. Within certain themes, multiple sub-themes also emerged. Representative quotes are also presented for each theme.

**Table 2:** Themes and representative quotes

Theme	Job role, setting of speaker
**Theme 1: Lack of family time or resources to adhere to FBT model**	
“Yeah, you can do it if you don’t have to work outside the home for 10 h a day in order to make ends meet, then you can easily shop for, cook and present and supervise three meals and three snacks a day for your child.”	Nurse practitioner, specialty eating disorders clinic
**Subtheme 1a: Engaging in FBT while working-full time is impractical for many caregivers, but quitting a job or taking a leave of absence may not be possible.**	
“It’s harder to get them good care. I think, you know, because of their support systems in a lot of ways, and their resources. And not because we’re asking them to pay for things but because if they’re…a parent is working, and unable to really supervise and bring them places, that creates a large barrier.”	Physician, community health clinic
“But yeah, to actually be able to sit and do meal support, a lot of times our parents are working full time at least and so the kids are going to school, school, in that situation, those kids probably aren’t going to schools that have a lot of extra support to deal with them.”	Therapist, community mental health clinic
**Subtheme 1b: Obtaining the amount of food necessary to refeed an adolescent with anorexia nervosa is a source of significant financial stress.**	
“Financial insecurity. So parents that really depend on food stamps or government assistance and maybe they can’t afford Ensure. Maybe they can’t afford the supplements that their child is choosing to eat.”	Social worker, specialty eating disorders clinic
“Yeah, so a lot of times our population were coming from lower socioeconomic status. So that was interesting to see they didn’t always have the means of getting the food that they needed, and that just always brought up a bunch of stressors within the family in general.”	Therapist, community mental health clinic
**Theme 2: Psychosocial barriers unrelated to FBT model that interfere with implementation**	
**Subtheme 2a: Psychiatric comorbidity**	
“And sometimes the sad truth is that we have to address the major mental illness or the behavioral problems. Sometimes like before we can make a whole lot of progress with the eating disorder. Except they go hand in hand because if you have a starving brain then you have a poorly emotionally regulated brain.”	Nurse practitioner, specialty eating disorders clinic
“Well so one thing that I think I see a fair amount of is…difficult home situations, not great support, and behavioral concerns that are contributory. So for example, a patient who is eloping and taking high risk behaviors and using drugs and doing other things, that’s contributing to her really poor nutrition and maybe body image too but body image is not the driving force, and they don’t really have a great support system at home to get them into the treatment that they need. That happens a fair amount with my patient population.”	Physician, community health clinic
**Subtheme 2b: Lack of outpatient FBT providers in the community**	
“I think there was a lack of step down for our population. I think in Rhode Island there’s not a ton of like, lower level of care eating disorder treatment so I think alright you’re working through FBT and now you’re ready to go to outpatient, and there wasn’t always the continuity there.”	Therapist, community mental health clinic
**Theme 3: Organizational barriers to implementation**	
“Some [providers] are not always so willing to do [FBT], and then maybe you have to make sure that everyone working in on that team understands that too, not just family but like the psychiatrist, the, if there’s a dietician involved, if you’re on a team, making sure that they, the other therapists on that team, and everything is really understanding of [FBT]… yes this is hard but we just have to get through this hump, instead of kind of pushing like “oh this shouldn’t happen, this treatment’s not working so you should try something else.””	Therapist, community mental health clinic
“We’re checking in [with the team] and we only have half an hour and we’re talking only about crisis flash issues that we really need to communicate about so it becomes a lot more like, “well 2 weeks ago, the dad came in and said…and then now we’re…” And it’s like, I just want to know what happened yesterday in that clinical appointment because we’re trying to figure out if she needs to go to [a partial hospitalization program], you know? So, it feels like we’re translating a lot to the supervising staff.”	Physician, specialty eating disorders clinic

#### Lack of family time or resources to adhere to the FBT model

Participants gave numerous examples of how a lack of time and resources made it difficult for families with lower incomes to adhere to the FBT model. Specifically, all of our participants spoke about the time and resources required to facilitate caregiver-led, high-calorie meals. Many participants reported that when FBT can be implemented with fidelity, they consider it to be the most effective treatment for adolescent AN, but they questioned whether most families presenting for treatment in community agencies would have the time and resources to implement it as designed. Participants noted that preparing, plating, and supervising three meals and multiple snacks per day put a tremendous amount of burden on the families with which they worked. These participants also specifically spoke to two subthemes (a) the ability of families to juggle full-time jobs along with participating in FBT and (b) the amount of money required to provide adolescents with the high calorie diets needed for regaining weight.

### Engaging in FBT while working full-time is impractical for many caregivers, but quitting a job or taking a leave of absence may not be possible

Participants reported that the demands of being an FBT caregiver often seemed inconsistent with full-time work outside of the home. Many noted that this was particularly the case for solo parents working long hours who did not have the benefit of a second income to rely on for support. One reported, *“There’s just no way that you’re going to be able to take the time to cook, to purchase the ingredients, cook the meal, sit your kid down, watch them eat the meal, for 3 meals and 3 snacks a day.”* (P5, Nurse practitioner, specialty eating disorder clinic). Although there was an emphasis on the time demands being especially impractical for low-income or solo parents, some participants also questioned whether FBT would be practical for any family with caregivers who worked many hours, regardless of income, *“And that also feels unfair to me, that we’re asking that of these families, whether they are under-resourced or whether they’re just stressed. Like I couldn’t do FBT in my house. It wouldn’t work!”* (P6, Physician, specialty eating disorder clinic). They also questioned the feasibility of the suggestion that a caregiver should consider taking a leave of absence from work to implement FBT, which is a part of the traditional FBT treatment manual, especially in the communities with which they work, *“It’s like the single mom… she knows that she’s supposed to do it. And…she can quit her job, or she can not do [FBT].”* (P6, Physician, specialty eating disorder clinic).

### Obtaining the amount of food necessary to feed an adolescent with anorexia nervosa is a source of significant financial stress for families with lower incomes

Participants reported that obtaining food was a barrier to treatment because food scarcity made parents less willing to fill an adolescent’s plate if they were not confident the adolescent would eat the food. *“A fair amount of our kids also have their meals come from food banks, so they don’t have a lot of food to be willing to put a big plate of food in front of a kid, just to throw it out…this is our food for the month. If she’s going to throw it away, I’m not going to give it to her because the other kid will eat it tomorrow.”* (P1, Therapist, community mental health clinic). This problem was compounded for families where immigration status made obtaining food more difficult, *“Again, especially not enough food in the homes, like for example, if you’re not an American citizen but you came here when you were 2 or 3 years old, and you’re 15 now, you don’t know your status so: Oh but my mom cannot apply for food stamps and mom doesn’t want to tell the kids…because guess what, you guys are not even American citizens”* (P3, Therapist, community mental health clinic).

#### Psychosocial barriers unrelated to FBT model that interfere with implementation

In addition to model-specific barriers, participants also identified that many families faced psychosocial barriers that made it more difficult to implement FBT. Many participants expressed that families presenting to community agencies were more likely to have problems aside from the eating disorder that had to be prioritized to ensure that the family could continue functioning, such as language barriers, immigration status concerns, and high-risk behaviors on the part of the adolescent, like running away from home. Aside from family functioning, participants also identified lack of adequate insurance coverage as a significant barrier to obtaining FBT in the first place. Two predominant subthemes also arose regarding psychosocial barriers: (a) psychiatric comorbidity and (b) the lack of FBT providers in the community.

### Psychiatric comorbidity

Participants noted that many adolescents in this population had other significant mental health and behavioral concerns that had gone untreated until the adolescent was medically compromised due to the eating disorder. Some participants reported that these problems made it difficult to focus on the eating disorder. *“The focus wasn’t always on the eating disorder. Whether it was like, trauma or, you know abuse in the home, or what have you. So I think the intensity of the population often got in the way of being able to implement [FBT] as it probably should be implemented.”* (P9, Therapist, community mental health clinic). Participants consistently reported that these other comorbidities made it difficult to know which concern to prioritize and some expressed disagreement with the general guidance of FBT that weight gain should be the primary focus of treatment. *“I’m like…but I also want to focus on the anxiety and the depression…I want to be able to do [FBT] but if I’m only going to be focusing on, okay, you have to gain weight, the kids are going to get bored. The kids are like okay, now I ate and now what?” (P2, Therapist, community mental health clinic).*

### Lack of outpatient FBT providers in the community

Participants expressed frustration with the difficulty of finding outpatient providers who specialize in FBT because of both low supply and problems with insurance coverage. Medical providers noted that they have had difficulty finding adequate outpatient therapy referrals for patients who they treated for medical management of AN, which led to compromises in care. In speaking about what they look for in a therapist for their patients, one physician noted, *“I hate to say that …we have a pretty low bar. Someone who knows the language…is able to kind of engage the family, and able to support the treatment strategy of the parents doing all of the work and the kid kind of just showing up and eating…I will say, in my experience there are very few therapists that are doing, like family-based, like with all the family in the room.”* (P6, Physician, specialty eating disorder clinic). Because of the time-limited nature of home-based treatment, therapists also expressed concern around continuity of care with families who completed FBT-HB and then needed to step down to outpatient therapy, given that most outpatient therapists in the community did not practice FBT.

#### Organizational barriers to implementation

Participants reported that it was difficult to communicate with other members of the treatment team when community providers were often spread across different organizations, *“I think the biggest challenge…is sometimes there’s a lack of communication. Or a lot of mixed messages that the family will get where we’ll be working on one thing [at our community organization] and then the dietician or the doctor that they’re seeing at the [medical] clinic will say something completely different.”* (P2, Therapist, community mental health clinic). One participant noted that the high staff turnover common in community mental health added to these challenges, *“Since the FBT program started … I think there’s been a lot of challenges around everybody feeling as though we’re all moving in the same direction…I think that’s partly because there’s been a lot of turnover in the staff, both in the staff, like in the supervising capacity and then also in the clinicians who are going out and doing the work.”* (P6, Nurse practitioner, specialty eating disorders clinic). Of note, this high turnover was compounded by the COVID pandemic, which was at its height when these interviews were conducted. Participants also described feeling stretched to fill multiple roles on their teams, which impacted their ability to provide FBT, *“Like there was supposed to be someone in the hospital and someone in the clinic, which was going to be me. But then the person in the hospital left, so I’m the only social worker covering both, and unfortunately things just…things get left undone or things fall through the cracks, so it’s not the best care, or like, sometimes it’s just mediocre care because there’s so many patients and there’s so much going on.”* (P8, Social worker, specialty eating disorders clinic).

## Discussion

This study presents an initial qualitative evaluation of the barriers to implementing FBT in community settings serving individuals from lower-income backgrounds. Guided by the Replicating Effective Programs Implementation Strategy (REP), which focuses on balancing fidelity and flexibility in the implementation of a health care intervention, we conducted interviews with therapists, medical providers, dietitians, and social workers who work with community organizations participating in our then upcoming hybrid pilot implementation-effectiveness trial of FBT-informed care delivered in the home. Identified themes from the interviews focused on the practical demands of a therapeutic model that places primary responsibility for adolescent weight restoration on the parents (e.g., the time and money required for parents to take on this role) as well as participants’ reticence to recommend FBT to their patients in light of these demands. Participants also expressed concern about the high level of comorbidity and acuity in this population making it difficult to prioritize eating disorder symptoms, and they described limited institutional resources and the resulting difficulty with communication between providers as another barrier.

Although participants identified a range of psychosocial and organizational barriers to FBT implementation, almost all the barriers presented regarding difficulty with the FBT model itself related to families not having the time and resources to implement FBT with fidelity. While it is possible that the high level of parental involvement required of FBT may be a key component to fostering the parental self-efficacy that is inherent in the efficacy of the treatment in fostering weight restoration [24], the results of this study indicate that many providers have concern about whether the families they serve have the resources to commit the requisite time to treatment. It is estimated that FBT requires a parent to spend more than 20 h per week directly caring for the adolescent with AN and working with the FBT therapist [25, 26]. For caregivers who have unpredictable schedules or work multiple jobs, this time commitment may not be feasible, and the suggestion to take a leave of absence from work is a privilege that most participants did not think was feasible for the families they worked with. Our team has worked to address this problem through the home-based care model, in which families are provided more intensive, accessible services than they may receive in outpatient settings, including meal support from therapists and school and medical appointment attendance. However, we recognize that this transfers the burden of care from the parents/caregivers to the FBT therapists.

It is important to note that, data collection for this study occurred during the height of the COVID-19 pandemic. As such, many parents were already tasked with working full-time jobs while also taking care of adolescents who were home 24 h a day, seven days a week due to social distancing restrictions. This is compounded by the fact that a sizeable portion of lower paying jobs were either considered essential, and required in-person work during the pandemic, or were eliminated due to social distancing requirements. As such, adolescents may have been left at home alone during the day or night when parents had to report to work, or parents may have been dealing with underemployment and had fewer resources at their disposable to support a sick child. It is possible that this influenced participant perceptions of the feasibility of tasking parents with the job of adolescent weight restoration on top of the tremendous burden that they already faced during COVID-19. Additionally, these challenges may have led therapists to assume more responsibility for meal preparation and coaching of the adolescent when parents were required to be at work, or were looking for work, and adolescents were participating in virtual schooling at home. At the same time, COVID exacerbated the high turnover and unfilled positions in the agencies with which we worked, which may have led to further burnout for participants and for families who lacked continuity of care.

Future work should examine provider perceptions of the burden of FBT now that most COVID-19 restrictions have been lifted. This would help to determine whether perceptions have changed as childcare and schooling structures have normalized and parents are no longer required to provide 24/7 care. With regard to FBT-HB specifically, future research should also address the sustainability of tasking community-based mental health workers, who are often underpaid and overworked, with being an in-home support for the multiple families with which they work. Although this concern is not unique to eating disorders treatment, it is nevertheless a challenge that we have continued to encounter throughout the pre-implementation and implementation phase of FBT-HB. Additionally, should FBT-HB prove to be effective in our formal pilot trial, more qualitative data are needed to understand the barriers and facilitators of delivering FBT in the home setting specifically, including family perspectives. These interviews are currently being planned by our study team.

In addition to concerns about the time required to adhere to FBT with fidelity, participants also perceived that food insecurity, or lack of access to a sufficient amount of food to allow the adolescent to gain and maintain a healthy weight, may serve as a barrier to engagement in FBT. An underweight adolescent with AN may require increasing caloric intake per day, particularly in the beginning of recovery, to regain weight [27], which can put significant stress on families living on a fixed income or food assistance programs. One possible avenue for alleviating this stress is through advocacy to include prescription meals and supplements, similar to programs that have been instituted for the treatment of type 2 diabetes [28], that are covered by insurance and provided to families with an adolescent with AN at a low cost. Although this would require creativity on the part of the caregiver to ensure that the adolescent was still a part of the family meal and ate some of the same foods as the family, this would allow the adolescent to have requisite amount of food without concern that other members of the family will go hungry as a result.

In addition to these challenges, as a part of the REP implementation framework, we specifically asked participants to reflect on institutional challenges that made implementation of FBT more difficult in community settings. These included high staff turnover (not only within the community agency, but within collaborating agencies participating in multidisciplinary care) and challenges coordinating regular communication between different types of providers. This turnover was likely compounded by the COVID-19 pandemic, when the health risk of going into a family’s home was much greater for therapists, especially those who were immunocompromised. The pandemic also likely influenced participants needing to serve in multiple roles when other team members were sick or had left their positions to take care of more acute needs. However, aside from the impact of COVID-19, our participants all functioned in settings where there may not be an integrated eating disorders program, complete with a team of therapists, medical providers (including psychiatrists), social workers, and nutritionists, among other roles. As such, communication problems between providers were likely compounded due to differences in institutional support of FBT and inconsistent expectations about how the multidisciplinary team should function. Although the lack of an integrated eating disorder team is not unique to lower income communities or to community-based agencies, integrated teams have been found to support better treatment outcomes for FBT [29], and this care integration is especially crucial for underserved patients [30].

In general, many of the barriers to FBT-informed care that the participants in our study identified are not necessarily unique to lower income, racial/ethnic minority populations. For example, Scarborough [26] also found that lack of evidence-based treatment options in the community and the large amount of time and resources required of parents were barriers to obtaining and conducting FBT-informed treatments for families of many different demographic presentations. However, one key difference is that participants in the present study noted that the families with whom they worked often did not have the option to work around these challenges (e.g., being able to take a leave of absence from work to supervise meals for the day). This is consistent with previous research which has found that therapists have adapted FBT for diverse populations by providing the adolescent with more responsibility for feeding in a shorter time frame due to lack of caregiver availability [18].

Strengths of the present study include the assessment of perspectives across a range of disciplines (e.g., medical doctors, clinical social workers, registered dieticians). This allowed us to understand a wide range of barriers that may impact families with lower incomes and the providers with whom they work. One important limitation of this study is that we assessed a relatively small, homogenous sample of providers working in the same local community. However, many of the themes that we identified overlap with other more theoretical papers outlining possible challenges with implementing FBT more broadly [26]. On the other hand, all of the therapists in the present study were familiar with FBT-HB while some of the medical providers were only familiar with the central tenets of FBT, which likely influenced their perception of the feasibility of implementation within the communities that they served. These differing experiences represent a limitation of the present study. Nevertheless, we attempted to lessen these differences by focusing on barriers to working with parents to assume responsibility of adolescent weight restoration, a key goal of both FBT and FBT-HB. Additionally, although our sample of primarily non-Hispanic, White women is consistent with the overall demographics of the eating disorder workforce in the United States [31], the inherent bias of the participants in this study must also be acknowledged, as we have presented data from a group of mostly White, highly educated individuals commenting on the experience of mostly racial and ethnic minority families with lower incomes. Both our future research and the patients and families with whom we work would benefit from more concerted efforts to diversify the eating disorders workforce and to include patient and family voices within the research that we conduct. Other limitations include a focus on barriers and not benefits of FBT. We acknowledge that a fuller exploration of benefits of FBT may have helped to solidify buy-in, especially as we attempt to bring in community organizations that may have been initially reticent to adopt FBT. Finally, it is important to note that this study was conducted in the United States, which has a third-party payor insurance system as well as limited assistance for parents who must take family/medical leaves of absence. As such, some of our findings may be less generalizable to other countries where there are greater social safety nets that may make FBT informed treatment in lower income communities more accessible.

Given that FBT is currently time intensive, which may impact its accessibility to many families with lower incomes, future mechanistic research is needed to understand which components of FBT are essential to the success of the treatment and which components may be forgone in populations that are unable to complete them. Since many of the findings from the present study focused on a lack of resources available to families to fully assume the caregiver role, especially early on during treatment, of particular importance is understanding whether the therapist, or other members of the treatment team in more intensive home-based treatment, or other family members or friends in traditional outpatient treatment may be able to support parents/primary caregivers in taking responsibility for feeding the adolescent without disrupting the efficacy of the treatment or outcomes post-treatment.

In sum, the present qualitative study underscores the need to maintain “flexibility within fidelity” [32] when implementing FBT with families from with lower incomes. It also emphasizes the need to understand the resources required of families, providers, and organizations to deliver FBT in an effective manner. Indeed, the voices of the participants included in this study speak to the need for considering whether adaptations should be made to FBT to meet the needs of a wide variety of families from different socioeconomic groups.

## Data Availability

Formal data sharing proposal and agreement forms for the study can be requested from first author Amy Egbert.

## References

[1] Arcelus J, Mitchell AJ, Wales J, Nielsen SJ (2011). Mortality rates in patients with anorexia nervosa and other eating disorders: a meta-analysis of 36 studies. Arch Gen Psychiatry.

[2] Auger N, Potter BJ, Ukah UV, Low N, Israël M, Steiger H (2021). Anorexia nervosa and the long-term risk of mortality in women. World Psychiatry.

[3] Huryk KM, Drury CR, Loeb KL (2021). Diseases of affluence? A systematic review of the literature on socioeconomic diversity in eating disorders. Eat Behav.

[4] Mitchison D, Hay P, Slewa-Younan S, Mond J (2014). The changing demographic profile of eating disorder behaviors in the community. BMC Public Health.

[5] Accurso EC, Buckelew SM, Snowden LR (2021). Youth insured by medicaid with restrictive eating disorders—underrecognized and underresourced. JAMA Pediatr.

[6] Moreno R, Buckelew SM, Accurso EC, Raymond-Flesch M (2023). Disparities in access to eating disorders treatment for publicly-insured youth and youth of color: a retrospective cohort study. J Eat Disorders.

[7] Lock J, Le Grange D, Agras WS, Moye A, Bryson SW, Jo B (2010). Randomized clinical trial comparing family-based treatment with adolescent-focused individual therapy for adolescents with anorexia nervosa. Arch Gen Psychiatry.

[8] Lock J, Le Grange D (2019). Family-based treatment: where are we and where should we be going to improve recovery in child and adolescent eating disorders. Int J Eat Disord.

[9] Lock J, Le Grange D. Treatment Manual for Anorexia Nervosa, Second Edition: A Family-Based Approach. Guilford; 2015. p. 321.

[10] Crone C, Fochtmann LJ, Attia E, Boland R, Escobar J, Fornari V (2023). The American Psychiatric Association Practice Guideline for the treatment of patients with eating disorders. AJP.

[11] Murray SB, Le Grange D (2014). Family therapy for adolescent eating disorders: an update. Curr Psychiatry Rep.

[12] Accurso EC, Mu KJ, Landsverk J, Guydish J (2021). Adaptation to family-based treatment for Medicaid-insured youth with anorexia nervosa in publicly-funded settings: protocol for a mixed methods implementation scale-out pilot study. J Eat Disord.

[13] Goldschmidt AB, Tortolani CC, Egbert AH, Brick LA, Elwy AR, Donaldson D (2022). Implementation and outcomes of home-based treatments for adolescents with anorexia nervosa: study protocol for a pilot effectiveness-implementation trial. Int J Eat Disord.

[14] Goldschmidt AB, Tortolani CC, Accurso EC, Dunbar EMP, Egbert AH, Donaldson D (2023). Adapting family-based treatment for adolescent anorexia nervosa delivered in the home: a novel approach for improving access to care and generalizability of skill acquisition. J Eat Disord.

[15] Astrachan-Fletcher E, Accurso EC, Rossman S, McClanahan SF, Dimitropoulos G, Le Grange D (2018). An exploratory study of challenges and successes in implementing adapted family-based treatment in a community setting. J Eat Disorders.

[16] Couturier J, Lock J, Kimber M, McVey G, Barwick M, Niccols A (2017). Themes arising in clinical consultation for therapists implementing family-based treatment for adolescents with anorexia nervosa: a qualitative study. J Eat Disorders.

[17] Dimitropoulos G, Freeman VE, Lock J, Le Grange D (2017). Clinician perspective on parental empowerment in family-based treatment for adolescent anorexia nervosa. J Family Therapy.

[18] Dimitropoulos G, Singh M, Sauerwein J, Pedram P, Kimber M, Pradel M (2024). Examining clinicians’ perceptions and experiences working with diverse families in family-based treatment: common adaptations and considerations for treatment engagement. Int J Eat Disord.

[19] Couturier J, Kimber M, Barwick M, Woodford T, McVey G, Findlay S (2018). Themes arising during implementation consultation with teams applying family-based treatment: a qualitative study. J Eat Disord.

[20] Kilbourne AM, Neumann MS, Pincus HA, Bauer MS, Stall R (2007). Implementing evidence-based interventions in health care: application of the replicating effective programs framework. Implement Sci.

[21] Deacon BJ, Farrell NR, Kemp JJ, Dixon LJ, Sy JT, Zhang AR (2013). Assessing therapist reservations about exposure therapy for anxiety disorders: the therapist beliefs about exposure scale. J Anxiety Disord.

[22] Farrell NR, Deacon BJ, Kemp JJ, Dixon LJ, Sy JT (2013). Do negative beliefs about exposure therapy cause its suboptimal delivery? An experimental investigation. J Anxiety Disord.

[23] Guest G, Namey E, Chen M (2020). A simple method to assess and report thematic saturation in qualitative research. PLoS ONE.

[24] Hughes EK, Sawyer SM, Accurso EC, Singh S, Le Grange D (2019). Predictors of early response in conjoint and separated models of family-based treatment for adolescent anorexia nervosa. Eur Eat Disorders Rev.

[25] Raenker S, Hibbs R, Goddard E, Naumann U, Arcelus J, Ayton A (2013). Caregiving and coping in carers of people with anorexia nervosa admitted for intensive hospital care. Int J Eat Disord.

[26] Scarborough J (2018). Family-based therapy for pediatric anorexia nervosa: highlighting the implementation challenges. Family J.

[27] Marzola E, Nasser JA, Hashim SA, Shih P, an B, Kaye WH (2013). Nutritional rehabilitation in anorexia nervosa: review of the literature and implications for treatment. BMC Psychiatry.

[28] Rabaut LJ (2019). Medically tailored meals as a prescription for treatment of food-insecure type 2 diabetics. J Patient Cent Res Rev.

[29] Pehlivan MJ, Miskovic-Wheatley J, Le A, Maloney D, Research Consortium NED, Touyz S (2022). Models of care for eating disorders: findings from a rapid review. J Eat Disord.

[30] Jones EB, Ku L (2015). Sharing a playbook: Integrated care in community health centers in the United States. Am J Public Health.

[31] Jennings Mathis K, Anaya C, Rambur B, Bodell LP, Graham AK, Forney KJ (2020). Workforce diversity in eating disorders: a multi-methods study. West J Nurs Res.

[32] Kendall PC, Gosch E, Furr JM, Sood E (2008). Flexibility within fidelity. J Am Acad Child Adolesc Psychiatry.

